# A context-free encoding scheme of protein sequences for predicting antigenicity of diverse influenza A viruses

**DOI:** 10.1186/s12864-018-5282-9

**Published:** 2018-12-31

**Authors:** Xinrui Zhou, Rui Yin, Chee-Keong Kwoh, Jie Zheng

**Affiliations:** 1 School of Computer Science and Engineering, Nanyang Technological University, Nanyang Avenue, Singapore, 639798 Singapore; 2 School of Information Science and Technology, ShanghaiTech University, 393 Middle Huaxia Road, Pudong, Shanghai, 201210 People’s Republic of China

**Keywords:** Encoding scheme, Influenza, Antigenicity, Classification

## Abstract

**Background:**

The evolution of influenza A viruses leads to the antigenic changes. Serological diagnosis of the antigenicity is usually labor-intensive, time-consuming and not suitable for early-stage detection. Computational prediction of the antigenic relationship between emerging and old strains of influenza viruses using viral sequences can facilitate large-scale antigenic characterization, especially for those viruses requiring high biosafety facilities, such as H5 and H7 influenza A viruses. However, most computational models require carefully designed subtype-specific features, thereby being restricted to only one subtype.

**Methods:**

In this paper, we propose a **C**ontext-**FreeEn**coding **S**cheme (CFreeEnS) for pairs of protein sequences, which encodes a protein sequence dataset into a numeric matrix and then feeds the matrix into a downstream machine learning model. CFreeEnS is not only free from subtype-specific selected features but also able to improve the accuracy of predicting the antigenicity of influenza. Since CFreeEnS is subtype-free, it is applicable to predicting the antigenicity of diverse influenza subtypes, hopefully saving the biologists from conducting serological assays for highly pathogenic strains.

**Results:**

The accuracy of prediction on each subtype tested (A/H1N1, A/H3N2, A/H5N1, A/H9N2) is over 85%, and can be as high as 91.5%. This outperforms existing methods that use carefully designed subtype-specific features. Furthermore, we tested the CFreeEnS on the combined dataset of the four subtypes. The accuracy reaches 84.6%, much higher than the best performance 75.1% reported by other subtype-free models, i.e. regional band-based model and residue-based model, for predicting the antigenicity of influenza. Also, we investigate the performance of CFreeEnS when the model is trained and tested on different subtypes (i.e. transfer learning). The prediction accuracy using CFreeEnS is 84.3% when the model is trained on the A/H1N1 dataset and tested on the A/H5N1, better than the 75.2% using a regional band-based model.

**Conclusions:**

The CFreeEnS not only improves the prediction of antigenicity on datasets with only one subtype but also outperforms existing methods when tested on a combined dataset with four subtypes of influenza viruses.

**Electronic supplementary material:**

The online version of this article (doi:10.1186/s12864-018-5282-9) contains supplementary material, which is available to authorized users.

## Background

In the immune system, antigen molecules are often specifically targeted by and bind with antigen receptors such as antibodies. It is an important mechanism of adaptive immunology in host organisms to defend against invading pathogens like influenza viruses. The capacity of an antigen in binding with the receptors is called antigenicity. Hemagglutinin (HA) and neuraminidase (NA) are so far the only two membrane proteins known to characterize the antigenicity of influenza viruses. Therefore, HA and NA are under constant antigenic drift pressure to escape the human immune system, as well as the flu vaccines. The selection of flu vaccines is mainly dependent on the antigenicity of influenza viruses. Therefore, the rapid identification of influenza antigenic variants is crucial for an effective vaccination program.

Serological diagnosis of influenza is usually conducted by hemagglutination inhibition (HAI) assays or micro-neutralization (MN) assays, serving as the gold standard for the antigenic correlations among antigens and antisera. Regulatory agencies, such as the World Health Organization (WHO) and Centers for Disease Control and Prevention (CDC), take the HAI assay titers of viruses as one of the primary measurements for vaccine efficacy, i.e. the ability of a vaccine to prevent disease in vaccinated individuals [1]. Thus, characterizing the antigenicity of a viral strain is crucial for predicting the vaccine efficacy. However, such experiments are labor-intensive, time-consuming and not suitable for early-stage detection. Compared with laboratory-based serological diagnosis, computational prediction of antigenic dissimilarity using viral sequences enables large-scale antigenic characterization of influenza viruses. Importantly, sequence-based computational methods make it possible to characterize the antigenicity of those highly virulent subtypes such as H5 and H7 influenza viruses, without requiring high biosafety levels.

Smith et al. pioneered the analysis of antigenic clusters of influenza A/H3N2 from 1968 to 2003, by using the method of metric multidimensional scaling (MDS) to map the viral strains on a 2D map and group them into 11 clusters [2]. Since then, researchers have made efforts to apply machine learning techniques to the antigenicity analysis. Most machine learning algorithms, however, require the input to be numeric vectors of equal length. Encoding the non-numeric dataset (e.g. protein sequences represented by letters) is, therefore, an important step for the performance of machine learning methods. Researchers have designed a variety of features to encode the viral sequences and then feed them into classification algorithms. For example, Liao et al. grouped amino acids based on their polarity, charge and aliphatic. Pairwise sequence comparisons were encoded into binary vectors according to the substitutions in the same or different groups. Regression models were then constructed to predict the antigenic distances from the binary vectors [3]. Liao et al. assumed that viral pairs with antigenic HAI titers larger than 4-fold have significant differences in antigenicity, and therefore should be treated as “variants” (i.e. distinct). Furthermore, Sun et al. extended the work by taking antibody binding sites into consideration. A bootstrapped ridge regression method was applied [4] and achieved an average prediction accuracy of 83% on an influenza A/H3N2 dataset. Du et al. calculated the differences in 12 structural and physiochemical features as a binary vector for each pair of HA sequences [5]. By integrating those features, they predicted the antigenic relationship of influenza A/H3N2 viruses with a Naïve Bayes classifier. To improve the prediction, Qiu et al. incorporated the structural context of the HA protein for influenza A/H3N2, reaching an accuracy of 87.5% [6].

A major limitation of the above-mentioned strategies is that they depend on subtype-specific features. Limited by the difficulty and cost in doing experiments with those highly pathogenic strains, the HAI datasets for H5, H7 and H9 subtypes are rather small. Only a few researchers endeavored to analyze the antigenicity of those subtypes computationally [7, 8]. Besides, the development of a universal flu vaccine, i.e. a vaccine providing durable protection against several strains, is a goal that has been long sought after. Although the universal vaccine might still be a long shot, finding the antigenic patterns shared by multiple influenza subtypes would be one step towards it. Peng et al. analyzed the sequence mutation patterns of nine representative HA subtypes on the HA1 protein, and they found that these HA subtypes share similar patterns of moving average position information entropy (MAPIE) [8]. This provided a basis for developing a universal computational model for predicting the antigenicity of influenza. They also proposed a regional band-based method to predict the antigenicity of influenza for diverse subtypes, but the accuracy was only 75% on the combined dataset of multiple subtypes of influenza viruses. Although the defined regional bands are independent of the viral subtype, some of them are hardly correlated with antigenic variation, as was reported by Lees et al. [9]. Insufficient conserved information about the antigenicity of influenza viruses could hamper the prediction. Transfer learning could shed light on addressing this issue. Many examples have justified the feasibility for transfer learning, i.e. applying the knowledge discovered from previous tasks to a target task with fewer high-quality training data [10, 11]. Given the possible shared sequence patterns of multiple influenza subtypes, it is also plausible to develop a framework to apply the knowledge learned in H1 and H3, where there are large qualified serological assays data, to other subtypes with limited data.

The performance of computational models mainly depends on two factors: the quality of the input, i.e. data representation and the learning algorithm. A representation which keeps more relevant information about the predicting target will benefit the performance of machine learning models [12]. In this paper, we propose a method called **C**ontext-**FreeEn**coding **S**cheme (CFreeEnS) to encode protein sequence pairs into a numeric matrix.

CFreeEnS takes advantage of rich information about the physiochemical and structural properties of amino acids. This encoding scheme keeps information about conserved properties of amino acids, which makes it possible for learning methods (e.g. random forest) to capture the cross-subtype antigenic pattern of influenza viruses. Using random forest classifier as a downstream learning method, the predicting accuracy on every subtype (A/H1N1, A/H3N2, A/H5N1 or A/H9N2) is over 85.0%. On the influenza A/H5N1 dataset, it reaches 91.5%. The results show that CFreeEnS (integrated with random forest) outperforms other methods that use carefully designed subtype-specific features. On the combined dataset, the average testing accuracy of CFreeEnS reaches 84.6%, higher than 75.1% of the regional band-based universal model [8]. Besides, we investigate the performance of CFreeEnS in transfer learning. Specifically, we use a testing dataset with a subtype of influenza A viruses different from the training dataset. The highest accuracy prediction accuracy is 84.3% when the model is trained on the A/H1N1 dataset and tested on the A/H5N1. The proposed CFreeEnS uses substitution matrices in the AAIndex database [13]. Then, we systematically evaluated the performance of all the available indexes. By analyzing the performance patterns of those indexes, we found several physiochemical and biochemical properties could be closely related to the antigenicity of influenza viruses, regardless of viral subtypes. The antigenic patterns of diverse influenza subtypes may give insights into conserved mechanisms of influenza virulence, thereby paving the way for a universal vaccine to provide protection against multiple subtypes of influenza viruses.

## Methods

**Fig. 1:**
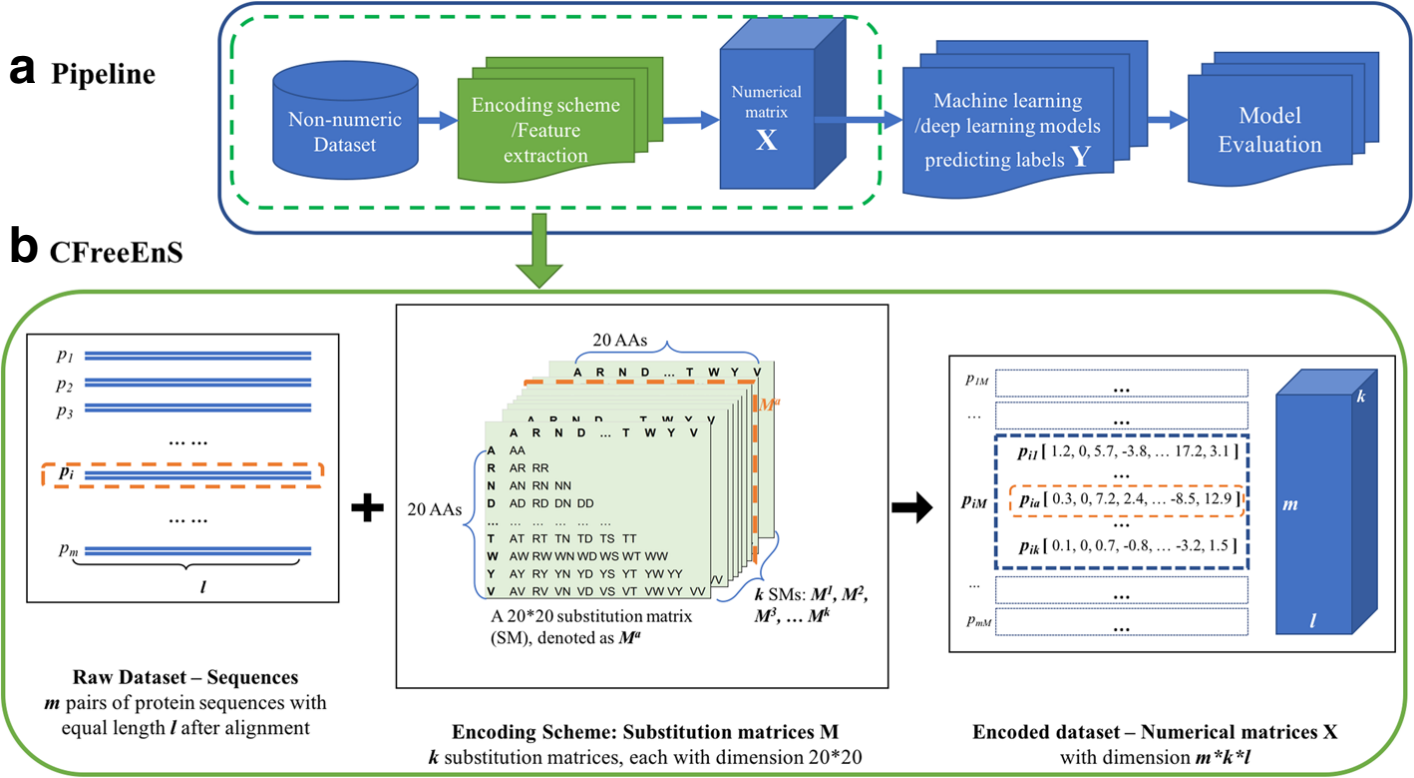
A pipeline for machine learning projects and illustration for CFreeEnS. **a** Encoding a non-numeric dataset into equal-length numeric vectors is necessary for both traditional machine learning models and deep neural networks. **b** CFreeEnS encodes *m* aligned protein sequence pairs of length *l* with *k* substitution matrices, resulting in a numeric feature matrix *X* with dimension *m*×*k*×*l*

Many machine learning algorithms, including deep neural network architectures, require an input of equal-length numeric vectors. A general pipeline for a machine learning project is shown in Fig. 1a. A non-numeric dataset should first be encoded into a numeric feature matrix *X* through some encoding scheme or handcrafted feature scores. Then, the numeric dataset *X* and label vector *Y* can be fed into machine learning models (e.g. deep neural networks) to minimize a loss function. The models should be evaluated with methods such as cross-validation for a separatetesting dataset. The performance of machine learning methods largely relies on the choice of data representation. Different representations can entangle and hide variant explanatory factors of the data.

In bioinformatics, encoding the symbolic amino acid data of protein sequences faithfully is an important step to improve the performance of model prediction. A good encoding scheme should preserve the information closely related to the problem. Although expert domain knowledge regarding the biological problem or the properties of proteins can benefit designing good encoding schemes, an encoding scheme requiring less expert domain knowledge and implementing more generic priors will help the automation of data-driven learning. The designing of an encoding scheme requiring less expert knowledge is also in line with the quest for artificial intelligence [12].

Here, we propose a context-free encoding scheme for pairwise protein sequences, named CFreeEnS, to convert protein sequence pairs into numeric vectors. CFreeEnS, based on the the published similarity matrices of amino acids, can capture the most important properties regarding the similarity of sequence pairs without designing features case-by-case. The representation of amino acids are constructed from amino acids level, involving different physiochemical and biological properties. Figure 1b shows how CFreeEnS works. For a batch of aligned protein sequences, suppose there are *m* sequence pairs with equal length *l* after alignment. Each pair *p*_*i*_, where (*i*=1,2,...,*m*), can be encoded using *k* substitution matrices $M^{a}_{20\times 20} (a=1,2,...,k)$. The score of *p*_*ia*_ at position *j* is calculated as [14]:

1 $$ \begin{aligned} {}p_{{ia}}[j] =\!\! \left\{ \begin{array}{ll} \!\!\!\left(M^{a}_{\phantom{\dot{i}\!}A_{1},A_{1}} \!\,+\, M^{a}_{\phantom{\dot{i}\!}A_{2},A_{2}}\!\right) \!\!- \!\!2M^{a}_{\phantom{\dot{i}\!}A_{1},A_{2}}, & \!\text{for}\ \!A_{1}!\,=\,gap\ \text{and}\ A_{2}!=gap \\ \lambda, & \text{otherwise} \end{array}\right. \end{aligned}  $$

where *A*_1_ and *A*_2_ are the amino acids at position *j* (*j*=1,2,...,*l*) of the two sequences respectively; $M^{a}_{x, y}$ is the score for amino acid *x,y* in substitution matrix *M*^*a*^. A penalty *λ* is encoded for gaps. Then, *p*_*ia*_ is a numeric vector with length *l*. Algorithm 1 shows how CFreeEnS encodes a protein sequence pair using one substitution matrix.


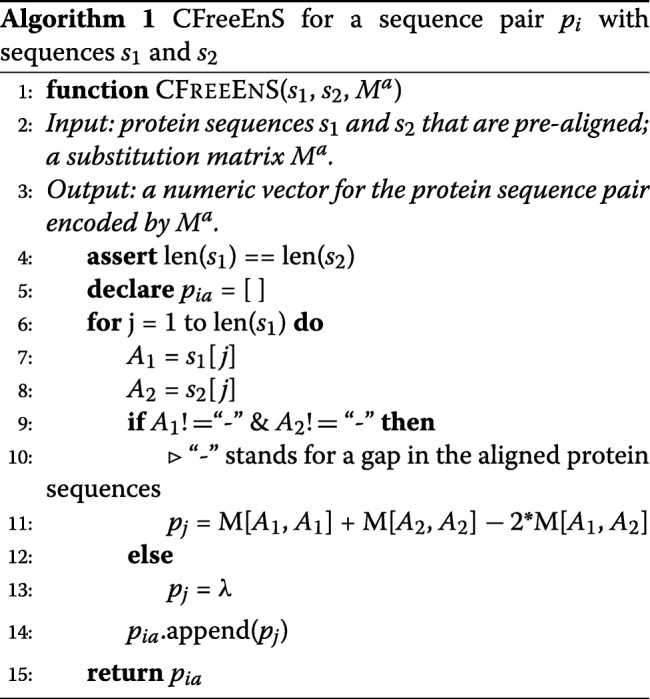


By stacking *k* such vectors [*p*_*i*1_,*p*_*i*2_,...,*p*_*ia*_,...,*p*_*ik*_], we can get the score matrix for sequence pair *p*_*i*_. Stacking the *m* instances together, an *m*×*k*×*l* scoring matrix *X* for the dataset is generated. Using CFreeEnS, a set of symbolic sequence pairs can be converted into numeric vectors with equal-length and then fed into machine learning models.

Currently, there are *k*=94 substitution matrices in the AAIndex database, preserving various physicochemical and biochemical properties of amino acid pairs [13]. This database provides an opportunity for systematically checking all substitution scoring matrices to select the most effective ones.

## Application

### Problem formulation

Sequencing has become cheap and fast. Therefore, we assume that HA1 protein sequences of the existing influenza viruses are available. Compared to viral sequences, the HAI data is much less, because it’s more expensive and time-consuming to obtain. The problem is how to accurately predict the antigenic distances based on the HA1 sequences of influenza viruses.

Instead of designing features for each subtype, we use CFreeEnS to encode protein sequences of viral pairs into a dissimilarity matrix *X*. The antigenic distances *Y* can be measured by the HAI assays. Referring to expert knowledge in this field, a distance threshold *θ* for judging two viral strains can be decided. Subsequently, the antigenic distances of viral pairs *Y* are discretized into a binary relationship vector *Y*^∗^ as illustrated in Eq. (2),

2 $$ Y^{*}(i,j) = \left\{\begin{array}{ll} 0, & \text{if}\ d(i,j)<\theta \\ 1, & \text{otherwise} \end{array}\right.  $$

where *d*(*i,j*) is antigenic distance between viral strain *i* and *j*; 0 represents “similar” and 1 represents “distinct” between the two viral strains *i* and *j*.

After encoding, we use a random forest, which is efficient and robust in handling thousands of input variables without manual selection of features [15], as a downstream learning method. The work is implemented using Python 3.6.4. A *RandomForestRegressor* in the *sklearn.ensemble* is used for training the model [16]. To avoid over-fitting, the maximum depth of trees is restricted to nine and all other parameters are set to default. The model is evaluated using metrics, including accuracy, precision, recall and F-score. Also, the learning curves regarding the mean-squared-log-error of training and testing datasets have been plotted to diagnose bias and variance of the computation model.

### Datasets

The proposed method for predicting antigenicity of influenza viruses does not rely on any subtype-specific feature. Therefore, it is universally applicable to all influenza subtypes. In this paper, the model is trained and tested on four subtypes which have drawn attention recently, namely A/H1N1, A/H3N2, A/H5N1 and A/H9N2.

#### Antigenic data

Antigenic HAI assay data of the four influenza viruses were collected and used to train computational models for predicting the antigenic distances of influenza viral pairs [8]. The Archetti-Horsfall distance (dAH) is taken as antigenic distance between a pair of viral strains [17], which has been reported to be more robust and less dependent on antigenic factors than other measurements [18]. The dAH between viral strains *i* and *j* is calculated in Eq. (3).

3 $$ dAH(i,j) = \sqrt{\frac{H_{{ii}}H_{{jj}}}{H_{{ij}}H_{{ji}}}}   $$

where *H*_*ij*_ is the HI titer of viral strain *i* relative to antisera raised against viral strain *j*. The antigenic distances of viral pairs *Y* are then discretized into a binary relationship vector *Y*^∗^ with a threshold of *θ*=4 [3] as illustrated in Eq. (2). The estimated antigenic distances $\hat {Y}$ vector can be inferred from *X* by training regression models, and then discretized with the same threshold to obtain the estimated binary relationship vector $\hat {Y}^{*}$.

Using the dAH measure, distances of 355, 791, 293 and 118 antigenic pairs were calculated for influenza A/H1N1, A/H3N2, A/H5N1 and A/H9N2 viruses, respectively. The percentages of distinct viral pairs in total viral pairs are listed in Table 1. The influenza A/H1N1 has approximately equal number of similar and distinct viral pairs, while the influenza A/H9N2 has more distinct pairs, around 68% in all the viral pairs. The imbalance between the similar and distinct pairs in the influenza A/H9N2 dataset may reduce the effectiveness of the predicting method. For the combined dataset, mixing antigenic data from all the four subtypes, the percentage of distinct viral pairs is 52% in all the viral pairs, which means the combined dataset has roughly balanced “similar” and “distinct” viral pairs.

**Table 1:** Datasets for training and testing the predicting model

Subtype	Number of sequences	T	D/T	HA1 lengths
H1N1	68	355	0.50	327
H3N2	621	791	0.47	329
H5N1	148	293	0.57	320
H9N2	29	118	0.68	317
Combined	866	1557	0.52	340

^1^T: Total number of viral pairs;

^2^D: The number of antigenic distinct viral pairs;

^3^Combined: The combined dataset of H1N1, H3N2, H5N1 and H9N2

#### HA1 protein sequences

The HA1 protein sequences, the immunologic part of HA protein, of those viruses involved in HAI assays were derived from the Influenza Research Database [19]. For subtype-specific predictive models, the HA1 sequences were aligned according to subtypes. The lengths of HA1 sequences are 327, 329, 320 and 317 for influenza A/H1N1, A/H3N2, A/H5N1 and A/H9N2 respectively. For a universal model, HA1 sequences of all the four subtypes were mixed before being aligned. The length is 340 after the alignment, which were conducted using MAFFT v7.245 with the FFT-NS-2 progressive strategy [20]. The antigenic data and HA1 sequences are publicly available in supplementary materials. Table 1 is a summary of the datasets for training and testing the computational model.

### Model evaluation

For each dataset, the model is trained and tested with 10-fold cross validation. Assessment of the performance is based on the average of the following evaluation metrics:

4 $$ Accuracy = \frac{TP + TN}{TP + FP + TN + FN}  $$

5 $$ Precision = \frac{TP}{TP + FP}  $$

6 $$ Recall = \frac{TP}{TP + FN}  $$

7 $$ F-score = 2*\frac{{{precision}} \times {{recall}}}{{{precision}} + {{recall}}}  $$

Here, *TP*, *TN*, *FP* and *FN* denote true positive, true negative, false positive and false negative in the confusion matrix obtained from *Y*^∗^ and $\hat {Y}^{*}$.

For a dataset of a single subtype, we use only one substitution matrix to encode the dataset. All the available 94 substitution matrices are used for evaluation. And then, those matrices resulting in the optimal predicting model with the highest accuracy are used to encode the combined dataset with various subtypes.

## Results

### Predictions on datasets with single subtype

**Fig. 2:**
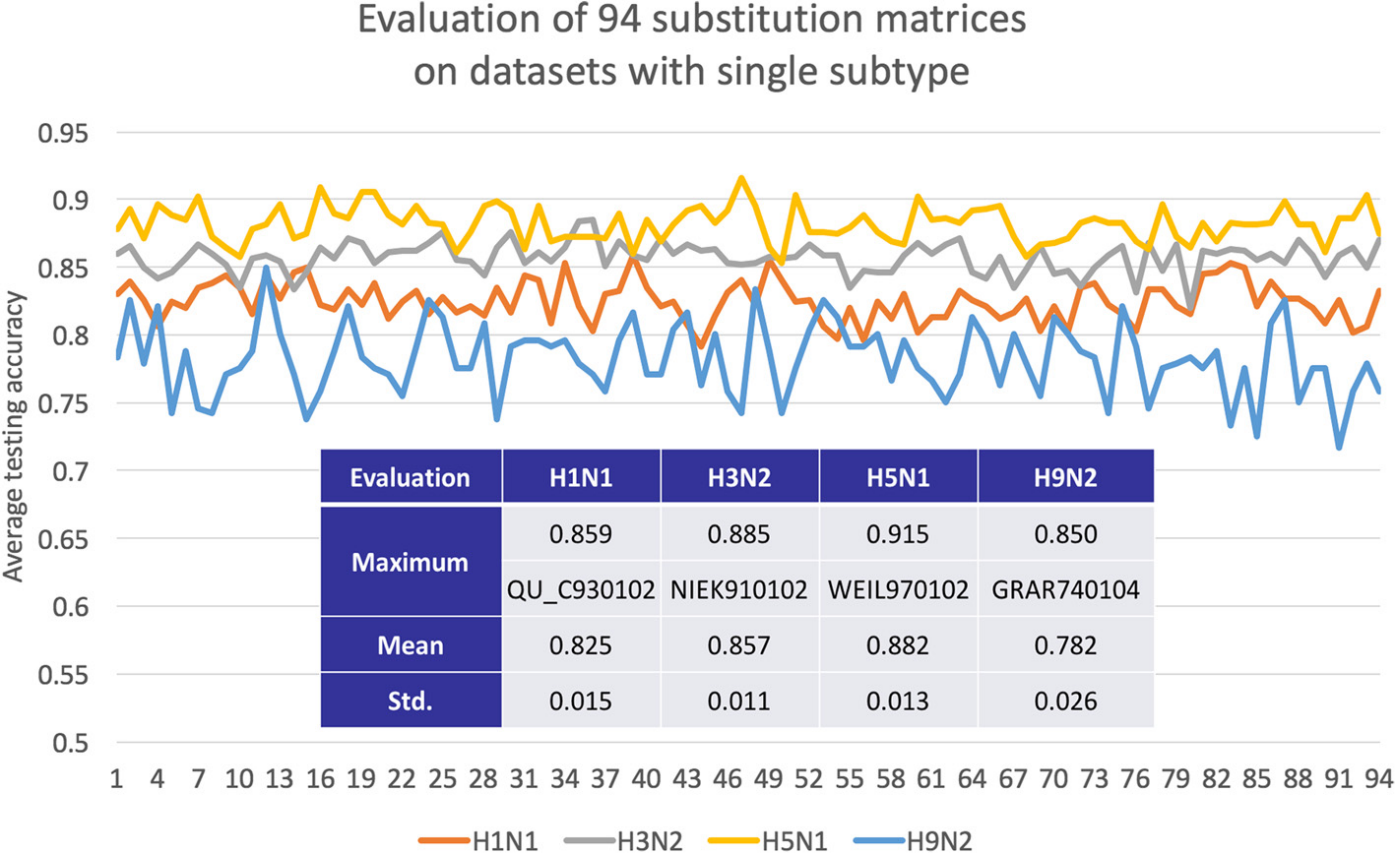
Evaluation of all substitution matrices on datasets of single subtype. The 94 substitution matrices have an average testing accuracy higher than 80% with small standard deviation, except on A/H9N2. Each dataset has a distinct substitution matrix resulting in the highest testing accuracy

For each dataset with a single subtype, namely A/H1N1, A/H3N2, A/H5N1 or A/H9N2, all the 94 substitution matrices were used to train a random forest with the same parameters. Each dataset has a distinct substitution matrix resulting in the highest testing accuracy, namely QU_C930102 for influenza A/H1N1, NIEK910102 for A/H3N2, GRAR740104 for A/H5N1 and WEIL970102 for A/H9N2. The results of testing accuracy are visualized in a line chart (Fig. 2). Overall, using only one substitution matrix to encode the dataset, the testing accuracy has small standard deviation (< 1.5%) in each dataset, except for A/H9N2. The strategy has the best performance on the A/H5N1 dataset with an average testing accuracy of 88.2% (± 1.3%), but the worst on the A/H9N2 dataset with the accuracy of 78.2% (± 2.6%). The imbalance in the A/H9N2 dataset with 68% distinct viral pairs could partly explain the lower performance.

The best predicting accuracy score for each subtype is greater than 85%, reaching 91.5% on the A/H5N1 dataset. Models obtaining the best performance are based on different substitution matrices, namely QU_C930102 for A/H1N1, NIEK910102 for A/H3N2, GRAR740104 for A/H5N1 and WEIL970102 for A/H9N2. In QU_C930102, the matrix was inferred from the contacts of main chain atoms [21]. NIEK910102 is a structure-derived correlation matrix considering the amino acid specific main-chain torsion angle distributions [22]. GRAR740104 combines mean chemical distances of properties: composition, polarity, and molecular volume [23]. WEIL970102 is a matrix obtained by subtracting the BLOSUM62 from the WAC matrix [24].

In addition, we compared the proposed encoding strategy CFreeEnS with the mutation-counts-based method proposed by Liao et al. [3] and regional band-based method proposed by Peng et al. [8] on the same datasets. It is worth noting that the methods use not only different encoding schemes, but also distinct training models. To demonstrate that our CFreeEnS is more accurate than the subtype-specific handcrafted ones, we also adapted the methods in literature by using random forest as the same training model, denoted as MutCounts and RegionBand respectively.

**Fig. 3:**
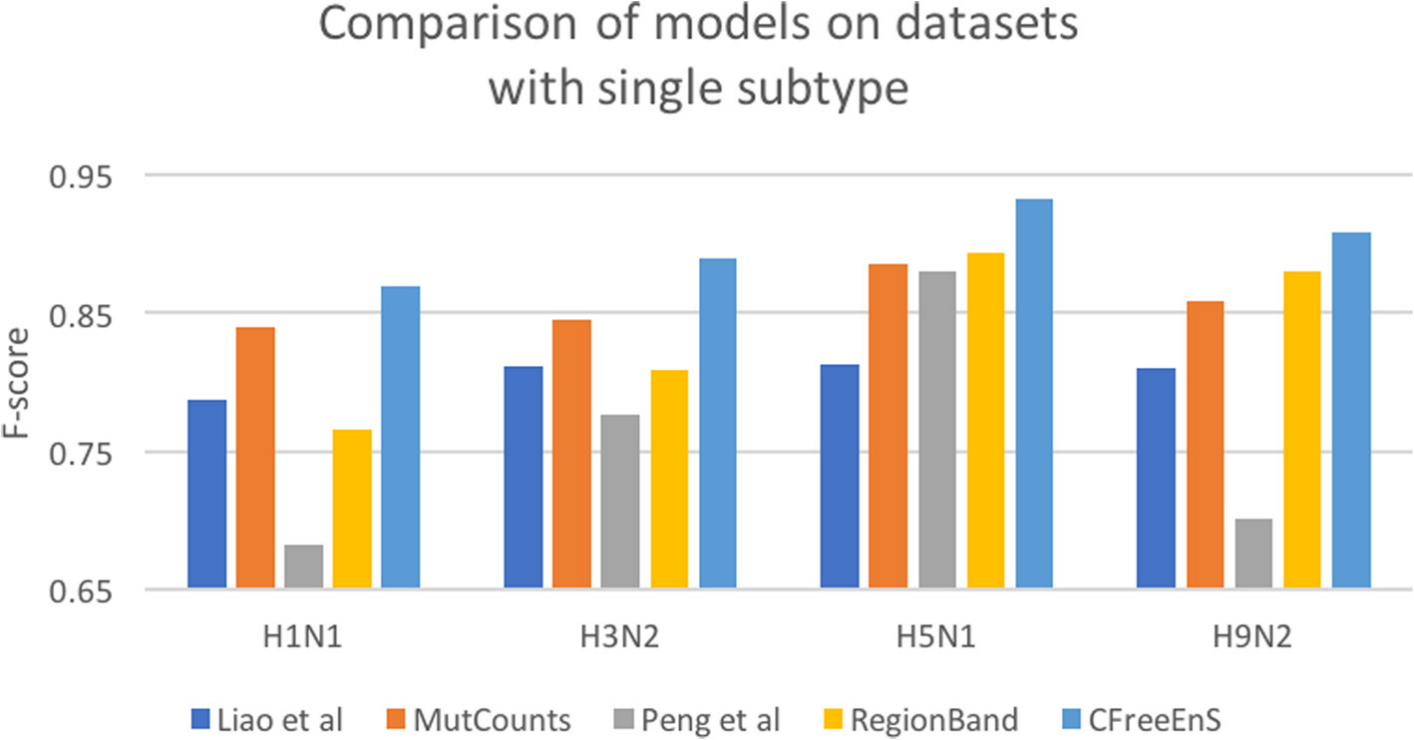
Comparing F-score of models on datasets with singe subtype influenza viruse

Figure 3 shows the comparison of F-score among five strategies on the four datasets with single-subtype influenza viruses. CFreeEnS obtains the highest F-score among the five strategies on all the four datasets (besides the combined dataset). Accuracy, precision and recall are also evaluated (Table 2). Although CFreeEnS sometimes ranks the second or third in precision or recall, it always obtains the highest accuracy and F-score. The experiments demonstrate that our proposed encoding scheme CFreeEnS outperforms subtype-specific features MutCounts and RegionBand in predicting the antigenicity of influenza viruses within the same subtype.

**Table 2:** Performance comparison among five strategies on four single subtype datasets

Dataset	Methods	Accuracy	Precision	Recall	F-score
H1N1	Liao et al.	0.742	0.717	0.877	0.788
	MutCounts	0.824	0.802	0.884	0.840
	Peng et al.	0.661	0.671	0.711	0.683
	RegionBand	0.706	0.669	red**0.901**	0.766
	CFreeEnS	^a^red**0.859**	red**0.856**	0.887	red**0.870**
H3N2	Liao et al.	0.784	0.748	0.891	0.812
	MutCounts	0.843	0.841	0.851	0.845
	Peng et al.	0.720	0.658	red**0.950**	0.777
	RegionBand	0.790	0.763	0.864	0.809
	CFreeEnS	red**0.885**	red**0.896**	0.882	red**0.889**
H5N1	Liao et al.	0.753	0.758	0.878	0.813
	MutCounts	0.863	0.859	0.915	0.885
	Peng et al.	0.846	0.857	0.908	0.880
	RegionBand	0.858	0.824	red**0.978**	0.893
	CFreeEnS	red**0.915**	red**0.903**	0.965	red**0.932**
H9N2	Liao et al.	0.708	0.816	0.819	0.810
	MutCounts	0.775	0.823	0.914	0.859
	Peng et al.	0.633	red**0.888**	0.601	0.702
	RegionBand	0.804	0.818	0.954	0.880
	CFreeEnS	red**0.850**	0.860	red**0.964**	red**0.908**

^a^The highest scores among five strategies on each dataset are colored red**red**

### Prediction on the combined dataset with diverse subtypes

For datasets with a single subtype, we traversed all the available substitution matrices. Each dataset has a distinct substitution matrix resulting in the highest testing accuracy, namely QU_C930102, NIEK910102, GRAR740104, and WEIL970102. The four substitution matrices, derived from different properties of amino acids, are selected as the optimal substitution matrices in predicting antigenicity of influenza viruses, denoted as CFreeEnS-4 to be distinguished from CFreeEnS which uses one substitution matrix. With CFreeEnS-4, the 866 viral pairs are encoded as a 866×4×340 matrix. To feed the data into machine learning models, it was flattened as a 866×1360 matrix, where the 4 feature vectors for each instance were stacked by column. Here, we used random forest with the same restrictions on maximum depth of trees, i.e. 9.

Table 3 presents the performance comparison among five strategies on the combined dataset. With 10-fold cross-validation, the average testing accuracy of CFreeEnS-4 on the combined dataset is 84.6%, higher than the second highest accuracy of 75.1% using the regional band-based method.

**Table 3:** Performance comparison among five strategies on the combined dataset

Dataset	Methods	Accuracy	Precision	Recall	F-score
Combined	Liao et al.	0.739	0.716	0.879	0.789
	MutCounts	0.698	0.675	red**0.944**	0.781
	Peng et al.	0.741	0.757	0.800	0.775
	RegionBand	0.751	0.723	0.912	0.807
	CFreeEnS-4	^a^red**0.846**	red**0.837**	0.900	red**0.867**

^a^The highest scores among five strategies on each dataset are colored **redred**

### Transfer learning: predicting the antigenicity of an emerging unknown subtype of influenza A virus

To check whether the knowledge gained in one subtype can be applied to the other subtype, we conducted transfer learning across subtypes. To be more specific, we trained a random forest using one subtype, and tested it on a different subtype of which not a single viral strain has been used in the training. For example, we trained a model on A/H1N1 dataset, and tested it on A/H3N2, A/H5N1, A/H9N2 datasets respectively.

**Fig. 4:**
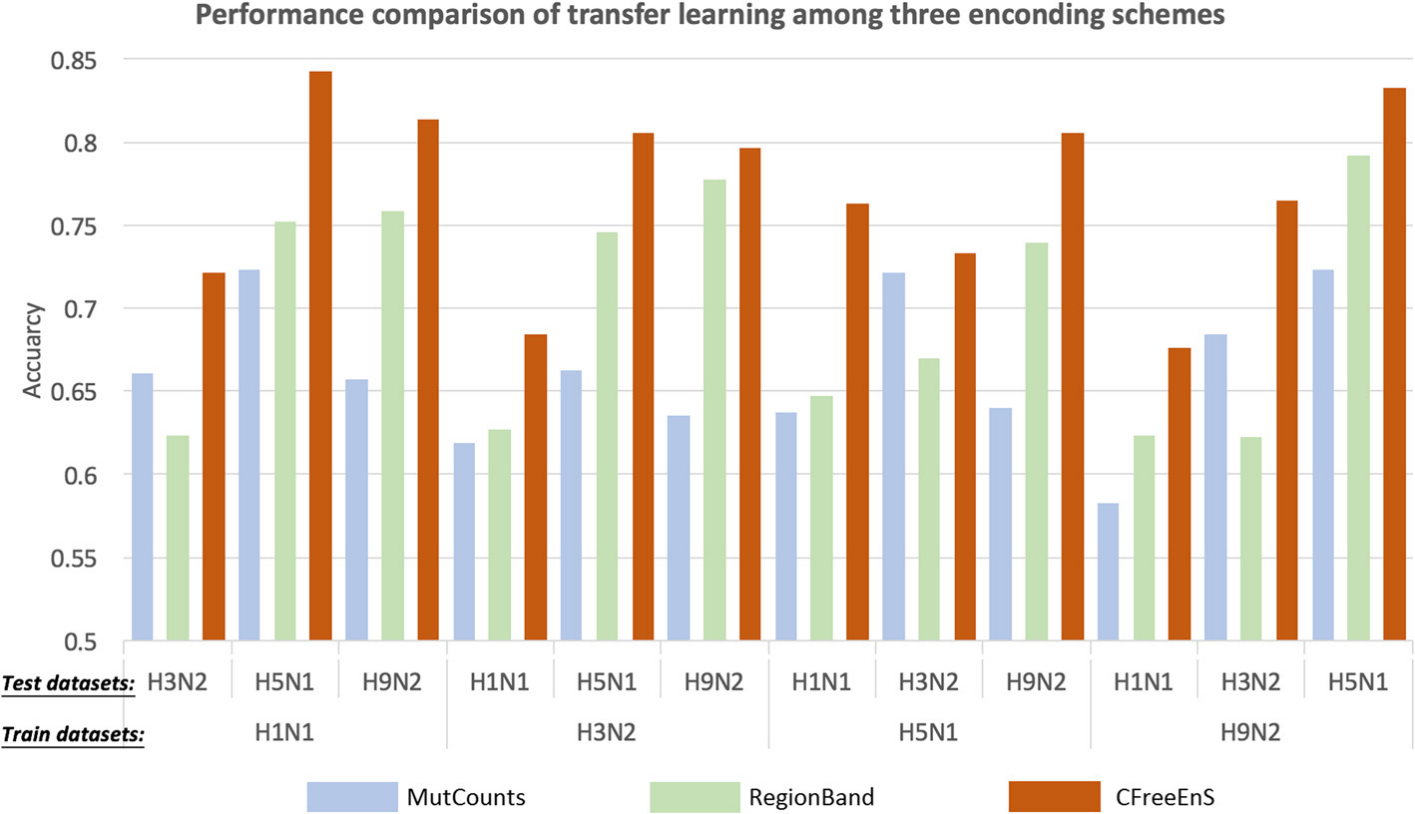
Accuracy scores of transfer learning using three encoding schemes: MutCounts, RegionBand and CFreeEnS. MutCounts: features that are used in the method proposed by Liao et al. [3]; RegionBand: features that are used in the method proposed by Peng et al. [8]. All the models use random forest as a downstream learning method

The accuracies of transfer learning using the three encoding schemes (i.e., MutCounts, RegionBand and CFreeEnS) are shown in Fig. 4. We can observe that CFreeEnS outperforms the other two encoding schemes in every experiment. The highest prediction accuracy is 84.3% when the model is trained on the A/H1N1 dataset and tested on the A/H5N1. The experiments of transfer learning indicate that CFreeEnS can encode generic properties conserved across subtypes. In addition, it gives a high accuracy in predicting the antigenicity of influenza A/H5N1 (83.3%) even with small training dataset like A/H9N2 (only 118 sequence pairs as training instances). The full result of comparison is available in Additional file 1. In some experiments, RegionBand has moderately better performance in recall. Overall, however, CFreeEnS has higher F-scores. Integrating the regional band-based handcrafted features into the encoding scheme might further improve the performance of prediction. Learning curves provided in Additional file 2 have shown that our models do not suffer the over-fitting problem.

## Discussion

The proposed CFreeEnS does not use any subtype-specific information, and thus can be applied to datasets with either one subtype or various subtypes. For a dataset with one subtype, one substitution matrix is enough to encode the dataset. All the available 94 substitution matrices are evaluated. Those with top ranking testing accuracy are used to encode the combined dataset with various subtypes.

The inconsistency of auto-selected substitution matrix indicates that different properties may dominate the viral antigenicity in different subtypes of influenza viruses. To improve the prediction in diverse subtypes, all those properties are taken into account to encode the combined dataset. The increases of predicting accuracy compared with MutCounts and RegionBand are 14.8% and 9.5% respectively, indicating that cross-subtype properties have been captured by the encoding scheme CFreeEnS. Further experiments on transfer learning have supported that the properties captured in one subtype of influenza can also work well in predicting the antigenicity of other subtypes of influenza.

## Conclusions

Our proposed encoding scheme CFreeEnS outperforms current methods that handcraft subtype-specific features when applied to predicting the antigenicity of influenza viruses, especially in the combined dataset with various subtypes. By systematically checking all the available substitution matrices, which consider different properties of amino acids, we find that properties related to the structures of amino acids or contacts between amino acids can help improve the prediction in the combined dataset. To be more specific, besides fundamental properties such as composition, polarity and molecular volume, information about contacts of main chain atoms and amino acid specific main-chain torsion angle distribution can help improve the predicting accuracy. This is consistent with our knowledge that different viral subtypes share major protein structures. The shared properties which affect the antigenicity of diverse influenza subtypes may give insights into the mechanisms of virulence of the influenza viruses. Another interesting finding is that the substitution matrices used in different subtypes are distinct. It suggests that the amino acid properties dominating the antigenicity of influenza viruses may vary from subtype to subtype.

The CFreeEnS, free from dependence on carefully designed features, is applicable to encoding different protein sequence pairs into a numeric matrix. It is promising for other applications in bioinformatics measuring the phenotype similarity from sequences, such as the neutralization escape of HIV-1 virus [25].

## Additional files

Additional file 1 Performances of three encoding schemes on transfer learning. A PDF document presenting full results, including accuracy, precision, recall and F-score, of transfer learning using the three encoding schemes (MutCounts, RegionBand and CFreeEnS). (PDF 115 kb)

Additional file 2 Learning curves. A PDF document presenting learning curves of random forest regressors trained on different datasets. (PDF 303 kb)

## Abbreviations

CDC: Centers for disease control and prevention
CFreeEnS: Context-free encoding scheme
dAH: The Archetti-Horsfall distance
HA: Hemagglutinin
HAI: Hemagglutinin inhibition
MAPIE: Moving average position information entropy
MDS: Metric multidimensional scaling
MN: Micro-neutralization
NA: Neuraminidase
WHO: World health organization
