# Supplementary material for: A context-free encoding scheme of protein sequences for predicting antigenicity of diverse influenza A viruses
Source: BMC Genomics. 2018 Dec 31;19(Suppl 10):936. doi: 10.1186/s12864-018-5282-9 (PMC6311925; doi:10.1186/s12864-018-5282-9)
Supplement: Supplementary file 1 — Performances of three encoding schemes on transfer learning. A PDF document presenting full results, including accuracy, precision, recall and F-score, of transfer learning using the three encoding schemes (MutCounts, RegionBand and CFreeEnS). (PDF 115 kb) [file 12864_2018_5282_MOESM1_ESM.pdf]

Additional file 1. Performances of three encoding schemes on transfer learning

TrainSet	TestSet	Method	Accuracy	Precision	Recall	F-score
H1N1	H3N2	MutCounts	0.660	0.649	0.758	0.697
		RegionBand	0.624	0.585	0.934	0.720
		CFreeEnS	*0.722	0.776	0.650	0.707
	H5N1	MutCounts	0.723	0.773	0.778	0.775
		RegionBand	0.752	0.726	0.961	0.826
		CFreeEnS	0.843	0.868	0.878	0.873
	H9N2	MutCounts	0.657	0.837	0.670	0.736
		RegionBand	0.758	0.770	0.959	0.854
		CFreeEnS	0.814	0.865	0.885	0.875
H3N2	H1N1	MutCounts	0.619	0.686	0.545	0.606
		RegionBand	0.627	0.626	0.774	0.692
		CFreeEnS	0.685	0.767	0.599	0.673
	H5N1	MutCounts	0.662	0.712	0.756	0.732
		RegionBand	0.746	0.738	0.909	0.815
		CFreeEnS	0.805	0.769	0.978	0.861
	H9N2	MutCounts	0.636	0.779	0.706	0.741
		RegionBand	0.777	0.789	0.952	0.863
		CFreeEnS	0.797	0.818	0.931	0.871
H5N1	H1N1	MutCounts	0.637	0.624	0.824	0.710
		RegionBand	0.647	0.626	0.861	0.725
		CFreeEnS	0.763	0.773	0.797	0.785
	H3N2	MutCounts	0.721	0.683	0.865	0.762
		RegionBand	0.670	0.621	0.929	0.744
		CFreeEnS	0.733	0.703	0.839	0.765
	H9N2	MutCounts	0.640	0.823	0.666	0.727
		RegionBand	0.740	0.759	0.949	0.843
		CFreeEnS	0.805	0.802	0.977	0.881
H9N2	H1N1	MutCounts	0.583	0.596	0.734	0.651
		RegionBand	0.624	0.601	0.909	0.723
		CFreeEnS	0.676	0.688	0.734	0.710
	H3N2	MutCounts	0.684	0.690	0.750	0.709
		RegionBand	0.622	0.585	0.934	0.719
		CFreeEnS	0.765	0.782	0.756	0.769
	H5N1	MutCounts	0.723	0.806	0.725	0.759
		RegionBand	0.791	0.757	0.977	0.853
		CFreeEnS	0.833	0.802	0.967	0.877

* The highest scores among three encoding schemes are colored red.
