# Supplementary material for: A context-free encoding scheme of protein sequences for predicting antigenicity of diverse influenza A viruses
Source: BMC Genomics. 2018 Dec 31;19(Suppl 10):936. doi: 10.1186/s12864-018-5282-9 (PMC6311925; doi:10.1186/s12864-018-5282-9)
Supplement: Supplementary file 2 — Learning curves. A PDF document presenting learning curves of random forest regressors trained on different datasets. (PDF 303 kb) [file 12864_2018_5282_MOESM2_ESM.pdf]

Additional file 2: Learning curves

A PDF document presenting learning curves of random forest regressors trained on different datasets.

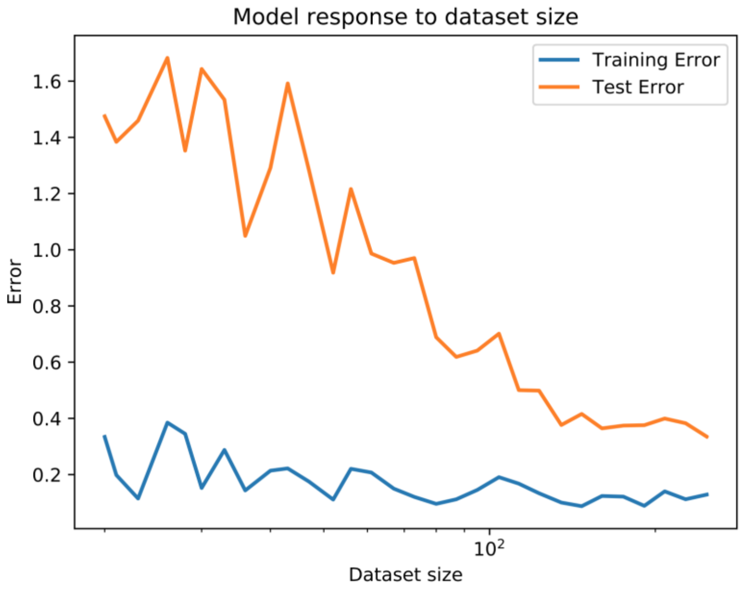

Figure S1 Learning curve of the Random forest regressor trained on H1N1 encoded using QU_C930102

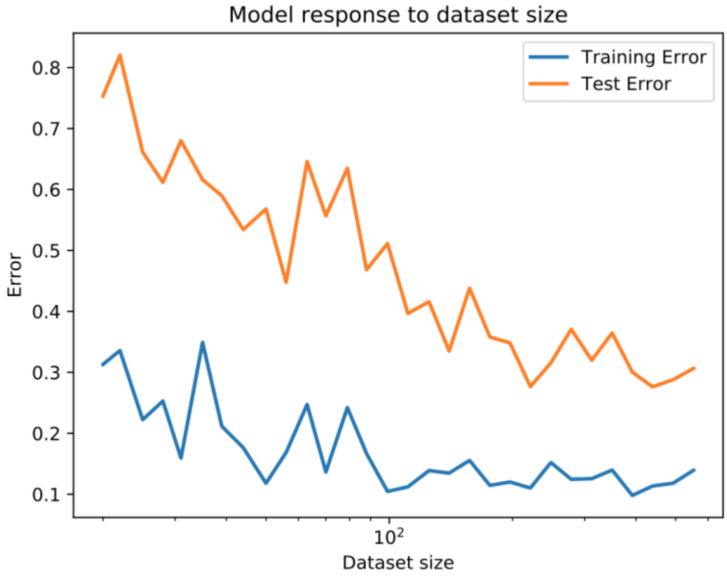

Figure S2 Learning curve of the Random forest regressor trained on H3N2 encoded using NIEK910102

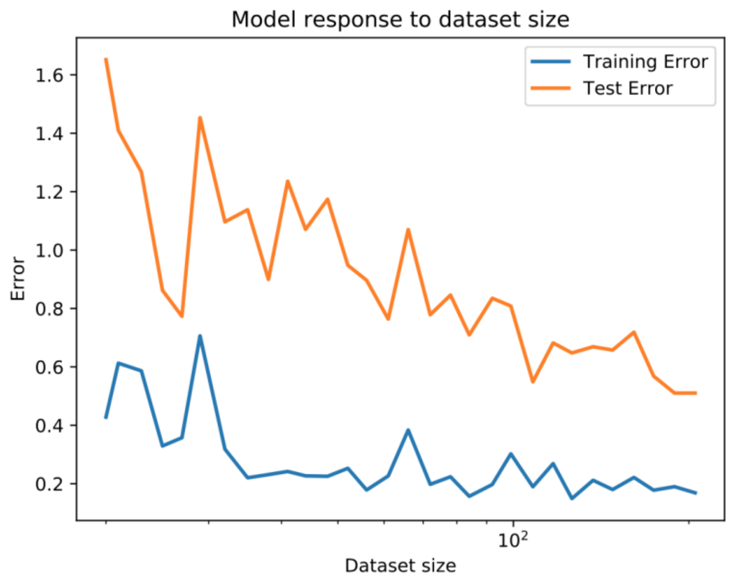

Figure S3 Learning curve of the Random forest regressor trained on H5N1 encoded using GRAR740104

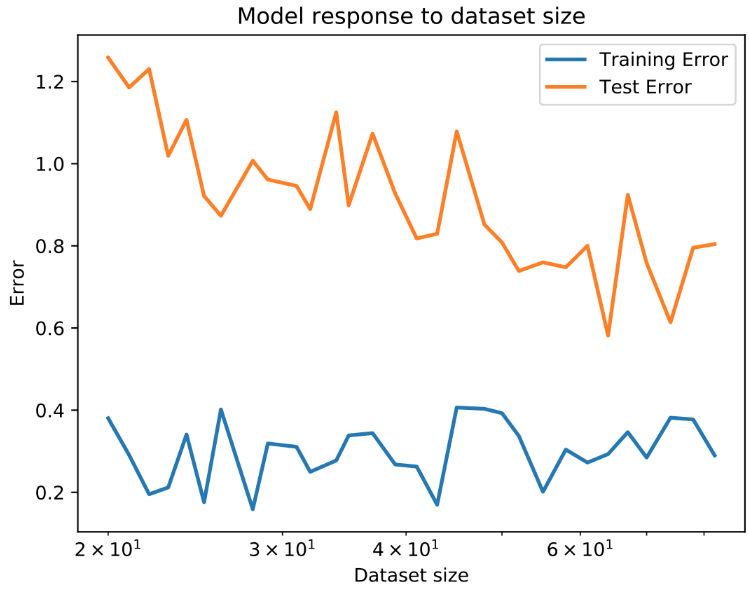

Figure S4 Learning curve of the Random forest regressor trained on H9N2 encoded using WEIL970102_0
